# Electric field-controlled transformation of the eigenmodes in a twisted-nematic Fabry–Pérot cavity

**DOI:** 10.1038/s41598-018-35095-y

**Published:** 2018-11-15

**Authors:** V. A. Gunyakov, I. V. Timofeev, M. N. Krakhalev, W. Lee, V. Ya. Zyryanov

**Affiliations:** 1 Kirensky Institute of Physics, Federal Research Center KSC SB RAS, Krasnoyarsk, 660036 Russia; 2 Laboratory for Nonlinear Optics and Spectroscopy, Siberian Federal University, Krasnoyarsk, 660041 Russia; 3 Institute of Engineering Physics and Radio Electronics, Siberian Federal University, Krasnoyarsk, 660041 Russia; 4 Institute of Imaging and Biomedical Photonics, College of Photonics, National Chiao Tung University, Guiren Dist., Tainan, 71150 Taiwan

## Abstract

The polarized optical states in the transmission spectrum of a twisted-nematic Fabry–Pérot cavity with the distinctly broken Mauguin’s waveguide regime have been theoretically and experimentally investigated. Specific features of the electric field-induced transformation of the polarization and spectral characteristics of eigenmodes of the neighboring series at the overlap resonant frequencies have been examined. It is demonstrated that the linear polarizations of eigenmodes at the cavity boundaries remain nearly orthogonal and their frequency trajectories reproduce the avoided crossing phenomenon. The experimental data are confirmed analytically and by the numerical simulation of light transmission through the investigated anisotropic multilayer with the use of a Berreman matrix method. The results obtained can be generalized to any materials with the helix response.

## Introduction

One of the promising directions in modern photonics is the development of controlled devices on the basis of structures with the permittivity periodically modulated in one, two or three dimensions on a spatial scale comparable to the light wavelength. Such structures are called photonic crystals (PCs)^1,2^. The Fabry–Pérot microcavities with the distributed Bragg mirrors, i.e., layered structures with the refractive index periodically changing in one spatial direction, are, in fact, one-dimensional PC structures with a defect layer. A specific feature of electromagnetic eigenstate spectrum in the layered structure is the presence of photonic band gaps (PBGs) almost totally reflecting the incident radiation^1–3^. The defect layer breaks the periodicity of dielectric properties and thereby leads to the localization of light with certain wavelengths inside the band gap.

The optical properties of the Fabry–Pérot cavity can be effectively controlled by using an electric field-sensitive medium as a defect layer. Here, the highly promising materials are liquid crystals (LCs), which exhibit a great variety of electrooptical effects useful for controlling the refractive index by changing the LC director configuration under low voltages^4^. Close attention of researches has been paid to the wave processes in optically anisotropic materials, including twisted-nematic LCs placed inside a Fabry–Pérot cavity. In such structures, the ease of controlling LCs by low voltages is combined with the high spectral resolution of the cavity^5–9^. This allows governing the intensity, phase, and polarization of the transmitted or reflected light^10,11^. It was analytically established that twisting of the optical axis of a nematic LC and the difference between the propagation constants of the extraordinary (*е*) and ordinary (*о*) waves in such a medium cause their coupling and form a new class of eigenmodes called twist extraordinary (*tе*) and twist ordinary (*tо*) waves^12^. These waves are elliptically polarized. The ellipticity of polarization is retained; the semimajor axis of the ellipse is directed along (*te*) or across (*to*) the local director. As was demonstrated using the theory of coupled modes, a pair of the *te* and *tо* waves at the same frequency is coupled by reflection in a twisted-nematic Fabry–Pérot cavity (TN-FPC). This coupling produces a cavity mode pair, *re* and *ro*. The polarization and, consequently, the mode type, *re* or *ro*, depend on the ratio between *te* and *tо* mode amplitudes^13^. In this case, despite the ellipticity of the cavity modes, they remain linearly polarized at the TN-FPC boundaries^14,15^. In a previous study^15^, the effect of mode coupling on the polarization states of eigenmodes of the TN-FPC containing a thin nematic layer with the distinctly broken Mauguin’s waveguide regime^16^ was investigated. The spectra were measured and calculated for the unpolarized incident light. It was shown that the device can be used as an electric field-controlled rotating linear polarizer. However, there are little-studied problems on specific features of the TN-FPC polarized transmission spectra, where peaks are accompanied by satellites. So, at first, the spectra seem to be random sets of peaks with arbitrary intensities. Experimental data that would reflect the correlation between the field-effect dynamics of the spectral positions of eigenmodes and change in their polarization state are lacking. In particular, it is important to clarify how the mode couplings manifest themselves at the field-effect transition through the Gooch–Tarry spectral point^17^, where the *te* and *tо* elliptic modes are maximally coupled. These problems have to be solved to regulate the concepts on the behavior of modes in twisted structures and optimize the structure of tunable TN-FPCs designed for telecommunication applications^18^. For this purpose, a modified experimental approach is needed.

The aim of this study is to investigate the spectral features of polarization components of the modes in a TN-FPC with a thin twisted-nematic layer within photonic band gap. We discuss the polarization and spectral behavior of selected modes in the vicinity of the Gooch–Tarry maximum under the field-effect dynamics. The spectral position of this point is governed by a low (~1 V) electric voltage and the spectra and polarization states of the *re* and *ro* cavity eigenmodes are detected by the rotating polarizer technique under their independent excitation. The experimental data are compared with the results of numerical simulation using the 4 × 4 transfer matrix method.

## Results and Discussion

**Figure 1:**
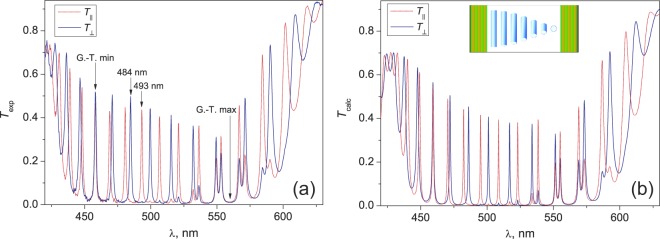
TN-FPC transmission spectra at the longitudinal (*T*_||_) and transverse (*T*_⊥_) polarizer orientations measured (**а**) and calculated (**b**) using the 4 × 4 transfer matrix method with regard to the mode decay (Im *n*_LC_ = 3.9·10^−4^). Arrows indicate the wavelengths corresponding to the Gooch–Tarry minimum (458 nm) and maximum (560 nm) conditions. Inset on the top shows a homogeneous twist-nematic structure.

The TN-FPC is a sandwiched structure (see in detail in the Methods) and consists of two dielectric multilayer mirrors separated by a thin twist-nematic liquid-crystalline film. Spectral properties of this device can be controlled with an electric field applied normal to the LC layer. The superimposed polarization components *T*_||,⊥_(λ) of the TN-FPC transmission spectrum measured in zero voltage are presented in Fig. 1a. Each component consists of two intervals that divide the PBG approximately in half. In the short-wave spectral range in the vicinity of a wavelength of λ_min_ = 458 nm, one can see a band of well-resolved peaks, which correspond to the *re* cavity modes in the *T*_||_-component and the *ro* modes in the *T*_⊥_-component. At the parameters of the investigated twisted-nematic structure, this wavelength corresponds to the Gooch–Tarry minimum condition^17^. At the wavelength of λ = 458 nm the values of the refractive indices *n*_e_ = 1.763 and *n*_o_ = 1.552 of the 5CB liquid crystal (*t* = 25 °C) and thickness *d* = 4.15 µm yield 2Δ*nd*/λ = 3.82. Thus, λ = 458 nm corresponds to the second Gooch–Tarry minimum. This condition simulates the Mauguin’s regime in the LC layer for the transmitted light linearly polarized along the director or orthogonally to it on the input mirror. In contrast to the Mauguin’s regime, the propagation of waves in the TN-FPC is not waveguide, since the modes in the bulk of LC remain elliptically polarized^13,19^. The wavelength λ_max_ = 560 nm shown by the arrow in Fig. 1a is a center of the mixed peak band. In particular, in the *T*_||_-component, along with the well-defined *rе* modes, the lower-intensity *ro* modes are observed as satellites and, vice versa, the *rе* modes are visible in the *T*_⊥_-component. At the parameters of the investigated twisted-nematic structure, the wavelength λ_max_ corresponds to the Gooch–Tarry maximum condition^17^. The electric field applied along the sample normal will unwind the nematic helix. The director field deformation is related to the weakening of the optical anisotropy of the LC medium, which, in turn, allows the spectral positions of modes to be controlled. For example, above some critical voltage applied to the sample, the mode λ_re_ = 493 nm will shift toward the mode λ_ro_ = 484 nm (Fig. 1a) and experience the avoided crossing phenomenon^6,8,13^. Figure 1b shows the calculated TN-FPC transmission spectrum. It can be seen that the experimental and calculated spectral positions of the cavity modes agree well within photonic band gap.

**Figure 2:**
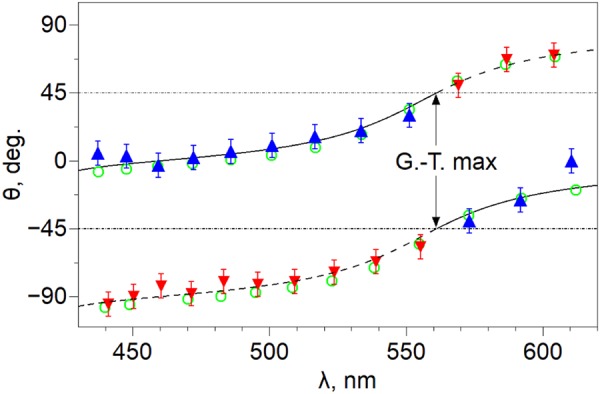
Angles θ of deviation of the linear polarization of the modes as a function of the LC director orientation at the output cavity mirror. Triangles show experimental values for the *ro* (Δ) and *re* (∇) cavity modes. Open circles show the numerical simulation data and the solid and dashed lines are built using Eq. (1).

The spectral features are explained by the essential difference between the states of polarization (SOP) of the optical modes in the vicinity of the Gooch–Tarry minimum and maximum. A parameter for estimating the SOP can be angle ξ or θ of the deviation of the linear polarization of the modes from the LC director on the input (ξ) or output (θ) cavity mirror, respectively. According to the approach described in ref.^19^, the angle ξ is determined as

1 $${\rm{\xi }}=\frac{1}{2}{\tan }^{-1}\,[-\frac{{\rm{\phi }}}{{\rm{\upsilon }}}\,\tan \,{\rm{\upsilon }}],$$

where ${\rm{\upsilon }}=\sqrt{{{\rm{\delta }}}^{2}+{{\rm{\phi }}}^{2}}$ is the twisted anisotropy phase, φ is the LC director twist angle, δ = Δ*ndk*_0_/2 is the anisotropy phase (angle), Δ*n* = *n*_e_ − *n*_o_ is the difference between the refractive indices of the *e* and *o* waves, and *k*_0_ is the wavenumber in vacuum. In addition, the analytical solution of Eq. (1) contains the LC frequency dispersion in the implicit form. For the investigated structure the angles ξ and θ are complementary. In particular, in the configuration presented in Fig. 8 below, the angle θ can be determined experimentally from the angle of deviation of the transmission direction of analyzer *А* from the *y* axis to the maximum transmission in this resonance. The θ values are assumed to be positive upon deviation of *А* to the positive direction of the *x* axis and negative upon deviation to the opposite side. Note that the rotation of the analyzer by 90° relative to the desired θ value leads to the quenching of the peak, which is indicative of the nearly linear polarization of the radiation at the cavity output. Figure 2 shows experimental and numerically simulated angles θ for all the resonant peaks of the TN-FPC spectra.

The θ(λ) functional dependence in Fig. 2 according to Eq. (1) allows us to follow the SOP evolution in the spectrum, starting from the λ_min_ wavelength. As expected, in the vicinity of the Gooch–Tarry minimum, *re* and *ro* modes are linearly polarized along the *x* || **n** (−90°) and *y* (0°) axes, respectively. As the λ value increases, the linear polarization smoothly and unidirectionally deviates from the coordinate axes up to critical angles of −45° for the *rе* modes and +45° for the *rо* modes at the Gooch–Tarry maximum point. It can be seen that the transition of this point leads to the change in the θ angle sign for the modes of both types. Thus, the modes, in fact, exchange by their SOP. Moving further to the long-wave region, the polarization directions monotonically approach the corresponding coordinate axes: θ → +90° (*x* axis) for the *rе* modes and θ → 0° (*y* axis) for the *ro* mode. The presented θ(λ) dependence elucidates the reasons for the essential difference between the structures of polarization components *T*_||,⊥_(λ) of the transmission spectrum (Fig. 1a) in the band of pure peaks and in the band of mixed ones. It is noteworthy that the above-mentioned trends to the TN-FPC mode SOP evolution in the spectrum are only determined by the strict alternation of the Gooch–Tarry extrema upon variation of the Mauguin’s parameter^16^ *u* ~ 2Δ*nd*/λ and, in this sense, are general. The number of bands in the PBG and their spectral positions can be different, since they are determined by the specific parameters of the investigated structure, including cavity thickness *d*, anisotropy value Δ*n*, and twist angle φ.

**Figure 3:**
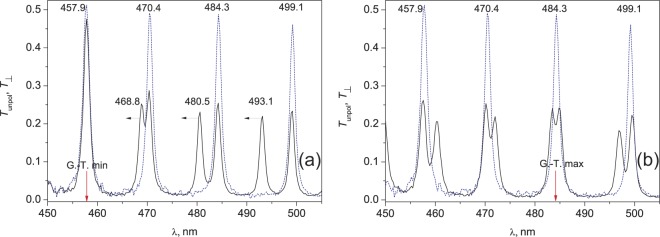
TN-FPC mode spectra measured for the unpolarized incident light *T*_unpol_ (solid lines) at (**а**) an under-threshold voltage of *U* = 0.74 V and (**b**) a voltage of *U* = 0.97 V at which the Gooch–Tarry maximum shifts to a wavelength of 484.3 nm from the initial position 560 nm (see Fig. 1a). The dashed line shows the spectrum of polarized *ro* modes (*T*_⊥_-component) measured in zero voltage.

Figure 3 presents experimental dependences of the polarized (*T*_⊥_-component) and unpolarized (*Т*) TN-FPC spectrum in the region of resolved peaks under applied voltages of 0.74 (Fig. 3a) and 0.97 V (Fig. 3b). The Freedericksz transition voltage is *U*_c_ = 0.76 V. For clarity, the field-effect dynamics of the spectral position of the modes is shown against the background of the spectra of the *T*_⊥_-component measured under zero voltage. The transmission peak position is determined by the cavity eigenmode frequencies. They satisfy the phase matching condition^15^, which requires the total phase incursion of eigenmodes for a cycle to be multiple of 2π:

2 $$2{\rm{\sigma }}\pm {{\rm{2sin}}}^{-{\rm{1}}}\,(\cos \,{\rm{\Theta }}\cdot \,\sin \,{\rm{\upsilon }})=2{\rm{\pi }}N.$$

here, the quantity 2σ = (*n*_e_ + *n*_o_)*k*_0_*d* is the mean mode phase for a cycle and the ellipticity parameter Θ = tan^−1^(φ/δ) reflects the smoothness of twisting relative to the nematic layer anisotropy value. The integer *N* = 1, 2, 3,… in Eq. (2) unambiguously determines the number of each resonant series from two peaks of close frequencies, which correspond to the *re* and *ro* mode. In Fig. 3a, one can see four such series. A remarkable property of the modes of one series is that they can cross with each other, i.e., resonate at the same frequency, only when the parameter υ amounts to an integer number of π. Coincidence of the *re* and *ro* modes at a wavelength of λ_min_ = 458 nm is an example of such crossing, which allows us to determine, according to the refractometric data on 5CB from ref.^20^, the number *N* = 30 of this series using Eq. (2). As the wavelength increases, the series number decreases by unity; thus, the series in Fig. 3a from the left to the right have the numbers *N* = 30, 29, 28, and 27. Another remarkable property of the twisted-nematic cavity is that the avoided crossing phenomenon can only be observed between crossed modes of the neighboring series with the numbers of different evenness^13^.

**Figure 4:**
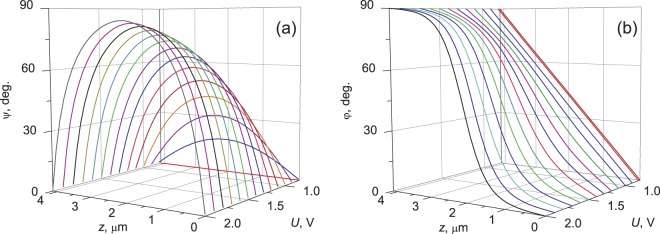
Simulated twisted nematic liquid crystal 5CB director field as function of the applied voltage is described by tilt angle ψ(*z*, *U*) (**a**) and twist angle φ(*z*, *U*) (**b**).

Simulated of the director field **n**(**r**) of the TN-layer of 5CB as a function of the applied voltage within 0.75 ÷ 2.0 V range is presented in Fig. 4. The electric field changes the distribution of the tilt ψ(*z*, *U*) and twist φ(*z*, *U*) angles of the local nematic director. It leads to the smooth shifting of the *rе* modes, while the position of the *ro* modes is almost field-insensitive. In particular, above the threshold voltage *U*_c_, the *rе* modes (468.8, 480.5, and 493.1 nm) shown by horizontal arrows in Fig. 3a start shifting from the initial position toward the nearest short-wave *ro* modes (457.9, 470.4, and 484.3 nm) of the neighboring series, forming mixed pairs with the latter (Fig. 3b). Under a certain voltage, each pair experiences the avoided crossing phenomenon, which indicates a new position of the Gooch–Tarry maximum for the structure under study. As an example, Fig. 3b shows the spectrum corresponding to the avoided crossing of the *rо* mode with 484.3 nm of the 28th series and the *rе* mode with 493.1 nm of the 27th series shifted toward the former under a voltage of *U* = 0.97 V. In the vicinity of the point λ = 484.3 nm, where the Gooch–Tarry maximum shifted at this voltage (shown by the arrow), the spectrum has the form of a doublet with the peaks symmetrically repulsed by ∼0.75 nm each relative to this point. Note that the mode spectra are analogous at the unpolarized incident light and in the presence of the polarizer for both *T*_||_ and *T*_⊥_ component. A further increase in the voltage to 1.05 V will lead to shifting of the Gooch–Tarry maximum to a wavelength of 470.4 nm and the occurrence of the avoided crossing phenomenon for the next mixed pair of modes, and so on.

**Figure 5:**
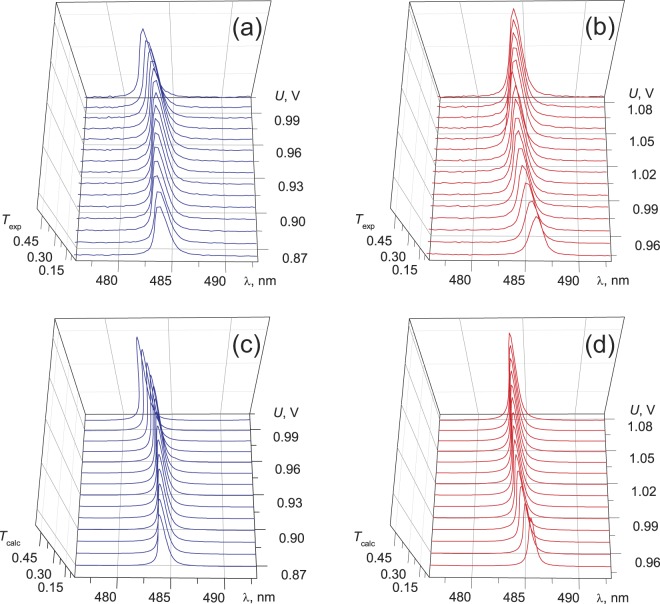
Experimental (**a**,**b**) and calculated (**c**,**d**) SOP spectra for the TN-FPC *ro* mode with 484.3 nm (**a**,**c**) and *re* mode with 493.1 nm (**b**,**d**) as a function of the applied voltage.

**Figure 6:**
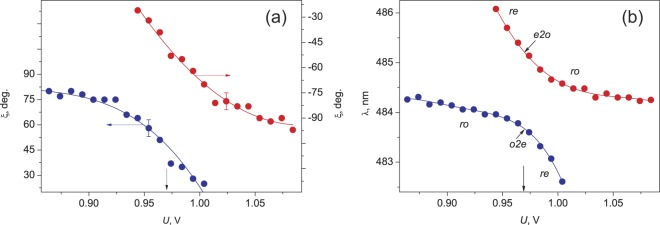
Experimental field-effect dependences of (**a**) the SOP and (**b**) spectral positions of the maxima of the modes with 484.3 nm (blue branches) and 493.1 nm (red branches) reproducing the avoided crossing phenomenon. The voltage *U* = 0.97 V is marked by the vertical line; solid lines show the interpolation.

It is interesting to follow the field-effect evolution of the SOP of a pair of modes, which experiences the avoided crossing phenomenon. To do that, the SOP spectra for a pair of modes with 484.3 and 493.1 nm were detected independently by the rotating polarizer method in the voltage range of 0.86 ÷ 1.10 V with a step of 0.01 V. In this method, for each voltage, a polarizer position is found at which the transmittance of the investigated resonant peak attains its maximum value *T*_max_. In this case, the angle between the transmission direction of polarizer *Р* and the *y* axis taken for the reference point corresponds to the angle ξ in Eq. (1). At the SOP orthogonality, the mode of the pair selected in such a way looks like a single peak without satellites. Figure 5 shows experimental and calculated the SOP spectra obtained by the rotating polarizer method for modes 484.3 nm and 493.1 nm as a function of the applied voltage. The dependences evidence for not only the mode orthogonality at the avoided crossing point, but also for the synchronous evolution of the eigenstates in the vicinity of this point upon monotonic variation in the voltage applied to the sample. The field-effect dependence of the angle ξ (Fig. 6a) measured with the polarizer set in a desired position before spectrum detection evidences for the not quite obvious fact of the SOP evolution at which the modes remain orthogonally polarized at least in the range of 0.94 ÷ 1.0 V. The unobviousness is caused by the different field-effect dynamics of the spectral positions of the modes with increasing voltage (Fig. 6b). In particular, upon approaching the voltages around *U* = 0.97 V, the *ro* mode with 484.3 nm, which is initially insensitive to the field, starts shifting to the blue spectral range, while the active *re* mode with 493.1 nm slows down. In this case, each mode in the pair resonates at its own frequency (Fig. 6b). Nevertheless, in view of the frequency closeness, the key parameters υ determining the direction of linear polarization of the cavity mode on the mirror^13,19^ differ insignificantly even at the end points of the λ(*U*) dependence in Fig. 6b, when the modes start diverging. In particular, at *U* = 0.94 V, the ratio between the anisotropy phases δ_rо_/δ_rе_ = (1 + Δλ/λ_ro_) and, consequently, between parameters υ_rо_/υ_rе_, differs from unity by only 0.4%. Here, Δλ = λ_re_ − λ_ro_ is the spectral interval between the combining modes. Such a discrepancy is noncritical for the ξ(υ) dependence of the polarization angles of the *rе* and *rо* modes in the range of small values of the υ parameter^13^, which results in the observed mode orthogonality effect at the essentially different field-effect dynamics of spectral positions of the modes. The specific feature of the behavior of the modes in the TN-FPC is that such independent characteristics as spectral position and polarization state are correlated. Comparison of the ξ(*U*) and λ(*U*) dependences shows that, e.g., the *ro* mode synchronously reacts to approaching the Gooch–Tarry maximum point by rotation of the angle ξ and shifting to the short-wave spectral region.

**Figure 7:**
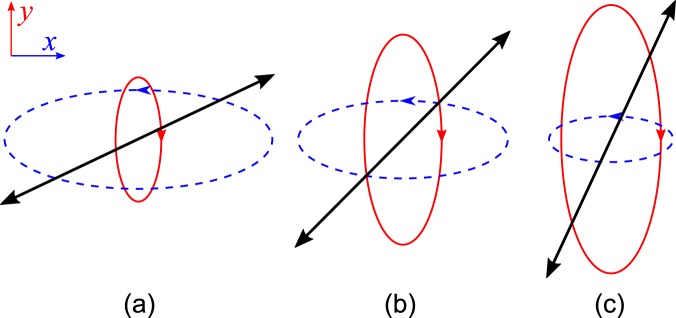
Transformation of the coupled elliptically polarized *to* (dashed lines) and *te* (solid lines) modes at the field-effect transition (а → b → c) through the Gooch–Tarry maximum spectral point. The nematic director **n** on the input mirror is aligned parallel to the *y* axes, light propagates along the *z* axes, and reciprocal arrows show the orientation of the linear polarization plane of the *ro* mode: the angle ξ relative to the *y* axes is (**a**) 65°, (**b**) 45°, and (**c**) 25°.

It is important that, in the case of orthogonal SOP of the modes in a mixed pair in the above-mentioned method for detecting the spectrum of a selected mode, the second cavity mode is blocked by the input polarizer, since both modes are linearly polarized at the boundaries. Nevertheless, the invisibility of the second mode does not affect the spectral position and polarization state of the investigated mode. In particular, at *U* = 0.97 V, the *ro* mode with 484.3 nm in Fig. 5a and *rе* mode with 493.1 nm in Fig. 5b occupy the same spectral positions as in the spectrum in Fig. 3b obtained by intensity equalization technique^15^. Thus, the independently detected modes appear repulsed by their virtual twins and thereby reproduce the trajectory corresponding to the avoided crossing phenomenon (Fig. 6). Coincidence of the mode trajectories obtained by different detection methods directly indicates their independence, despite the matched rearrangement of the polarization angles ξ under the action of the electric field. Indeed, the cavity modes represent a mixture of *te* and *to* waves in a certain ratio and do not couple, according to the definition 12. As an example, Fig. 7 shows transformation of the SOP resonant peak at the frequency of the *ro* mode combined from two elliptically polarized waves under the action of electric field when the resonance is not excited at the *re* mode frequency.

The observed rotation of the linear polarization plane indicates that upon approaching the Gooch–Tarry maximum, the coupling between elliptical waves strengthens and a periodic energy flow from the *to* to *te* wave and back increases. The resulting phase shift, which moves the resulting mode frequency to the blue spectral range, grows. The transition through the Gooch–Tarry maximum leads to the transformation *ro* → *o2e* → *rе* and the short-wave mode becomes field-sensitive (the lower branch in Fig. 6b). The synchronous character of the mixed pair evolution suggests the analogous transformation *rе* → *e2o* → *ro* of the SOP; therefore, the long-wave *rе* mode, which was earlier field-sensitive, transforms to the *ro* mode and occupies a fixed spectral position, in which the *ro* mode of the neighboring series was localized at the lower voltage (the upper branch in Fig. 6b).

Electric field-controlled transformation for polarization rotation is realized within peak profile of *ro*-modes. A high-speed defect mode switching in optical cavity with a nematic LC can be realized using a narrow peak schift and a fast part of the molecular orientation^21,22^. The response time of the presented rotation is expected about of milliseconds and can be improved to microseconds for ferro- or antiferroelectric liquid-crystal materials^23^ and has a potential for submicrosecond response^24,25^. We anticipate the presented polarization rotation principle operation for other helix materials with faster response in expense of the cavity thickness or absorption. The LC material extinction is critical to obtain cavity mode with high quality factor and sharp linewidth. The quality factor falls down for plasmonic materials and anisotropic metamaterials that provide stronger optical response, for example, in steering absorption^26^ and polarization properties^27^, as well as optical harvesting^28,29^.

## Conclusions

The polarization components of the spontaneous TN-FPC transmission spectrum with the distinctly broken Mauguin’s waveguide regime were experimentally and theoretically investigated. The correlation between the polarization and spectral characteristics for both the *rе* and *rо* modes at the field-effect transition through the Gooch–Tarry maximum critical point was demonstrated. The observed double response of the spectral peaks to the electric field-induced change in the phase shift between the elliptic waves forming the cavity mode is typical of the TN-FPC. At the critical point, each cavity mode transforms to the opposite one. In this case, the linear polarizations of the *rе* and *rо* modes at the TN-FPC boundaries remain nearly orthogonal and the trajectories of their superimposed frequencies reproduce the avoided crossing phenomenon observed under sample illumination by the unpolarized light. It was established that the mode transformation accompanied by the change in both mode polarization and spectral position is only determined by the mode coupling force of the elliptic waves and is independent of the excitation of the other mode in the cavity. The experimental results were confirmed analytically and by the numerical simulation of light transmission through the investigated multilayered structure using the 4 × 4 transfer matrix method. Diverse applications of the examined twisted cavity are anticipated in sensing, filtering, switching, and optical modulation in photonic, optoelectronic and telecommunication devices with the advantage of high resolution in both wavelength and polarization. We stress that the reported results can be generalized to materials with the fastest response and any helix structures^30^.

## Methods

**Figure 8:**
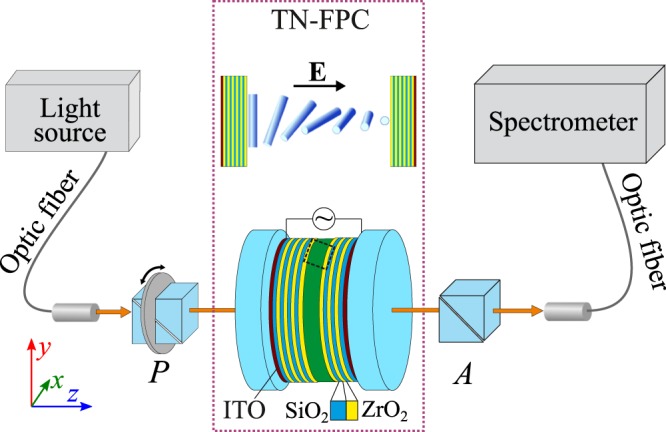
A schematic view of the experimental setup. The ZrO_2_/SiO_2_ multilayer mirrors of the TN-FPC are formed on the substrates with transparent ITO electrodes. The cavity is filled with the 5CB twisted-nematic LC disturbed by applied voltage (inset on the top). Polarizer *P* and analyzer *A* are Glan prisms.

The SOP of transmission peaks in the TN-FPC spectrum with and without control electric field were experimentally studied on a setup schematically shown in Fig. 8. The cavity with the distributed Bragg mirrors had the (ZrO_2_/SiO_2_)^5^ZrO_2_ (TN) ZrO_2_(SiO_2_/ZrO_2_)^5^ layered structure. The ZrO_2_ and SiO_2_ layers alternately deposited onto fused quartz substrates had refractive indices of 2.04 and 1.45 and thicknesses of 55 and 102 nm, respectively. The transmission spectrum of such a structure is a PBG in the spectral range of 425–625 nm with a set of resonant peaks corresponding to the modes localized on the twisted-nematic defect layer (Fig. 1). Thin indium tin oxide (ITO) electrodes predeposited onto quartz substrates made it possible to apply an electric field along the mirror surface normal. A gap between the mirrors with an actual thickness of *d* = 4.15 µm was filled with a 4-*n*-pentyl-4′-cyanobiphenyl (5CB) nematic LC. To form the twisted structure of LC director **n**, the mirrors were coated with polyvinyl alcohol (PVA) films and then unidirectionally rubbed. The crossed directions of rubbing the output and input cavity mirrors, where the director **n** is parallel to the *x* and *y* axes of the laboratory system of coordinates (*x*, *y*, *z*), respectively, ensured homogeneous twisting of the nematic director **n** across the LC layer by an angle of φ* = *90°.

The peculiarities of cavity assembly with regard to the features of the rubbed polymer films used to the planar alignment of the LC director with a slight surface pretilt^31^ formed a uniform right-handed twisting of the nematic structure. An ac electric field with a frequency of 1 kHz was applied to the sample to ensure smooth untwisting of the director **n** until quasi-homeotropic alignment (twist-effect). Transmission spectra of the TN-FPC were recorded on an Ocean Optics HR4000 spectrometer under polarized and unpolarized illumination at a fixed sample temperature of *t* = 23.5 °С. There are four (i–iv) principal configurations of the setup to study polarized optical states of the TN-FPC modes. (i) A single polarizer *P* was used to detect polarization components *T*_||,⊥_ of the transmission spectrum (Fig. 1). Here subscripts (||) and (⊥) indicate the parallel and perpendicular orientations of *P* relative to the **n** direction on the input mirror, respectively. (ii) A single analyzer *А* placed after the sample served to determine the mode polarization angles θ at the cavity output for all resonance peaks of TN-FPC spectra (Fig. 2). (iii) Unpolarized incident light was used to demonstrate the schift of the spectral positions of the modes with turning voltage on (Fig. 3). (iv) Finally, the rotating polarizer technique was used to determine the evolution of the SOP of the cavity eigenmodes depending on the applied voltage (Figs 5 and 6). The polarizers used are Glan prisms equipped with a dial and both can freely rotate in the (*x*, *y*) plane. Radiation was introduced in a sample and extracted from it using optical fibers.

The simulations are carried out using MATLAB to verify the observed results from the experimental spectra. None of the calculated values in space depends on *x*,*y*-axis, so the simulation is one-dimensional. In the first step the nematic orientational structure within the cell is calculated by means of the free energy minimization with rigid anchoring potential. The Frank elastic energy density *f*_k_ is expressed as

3 $${f}_{k}=\frac{1}{2}{k}_{11}\,{(\nabla \cdot {\bf{n}})}^{2}+\frac{1}{2}{k}_{22}\,{({\bf{n}}\cdot \nabla \times {\bf{n}})}^{2}+\frac{1}{2}{k}_{33}\,{({\bf{n}}\times \nabla \times {\bf{n}})}^{2}.$$

here **n** is the director, *k*_11_, *k*_22_ and *k*_33_ are the splay, twist and bend elasticity coefficients, respectively. At a fixed voltage the total electric contribution to the free energy density *f*_e_ is expressed as^32^

4 $${f}_{e}=\frac{1}{2}{\bf{D}}\cdot {\bf{E}}-{\bf{D}}\cdot {\bf{E}}=-\,\frac{1}{2}{\bf{D}}\cdot {\bf{E}}=\frac{1}{2{\varepsilon }_{0}}\,\frac{-{D}_{z}^{2}}{({\varepsilon }_{\perp }\,{\cos }^{2}\psi (z)+{\varepsilon }_{||}\,{\sin }^{2}\psi (z))}.$$

**E** is the vector of electric field applied to the LC layer, **D** is the vector of electric induction in the bulk of the LC, ψ(*z*) is the polar angle of the LC director deflection from the substrate plane, ε_⊥_ and ε_||_ are the LC permittivities transverse and longitudinal relative to the director. The electric induction **D** is constant across the cell as long as the divergence of the electric displacement is zero. The total electrostatic energy *F*_e_ of a cell can be expressed by voltage *U* as the following:

5 $${F}_{e}={\int }_{0}^{d}{f}_{e}dz=\frac{-{\varepsilon }_{0}{U}^{2}}{2{\int }_{0}^{d}{({\varepsilon }_{\perp }{\cos }^{2}\psi (z)+{\varepsilon }_{||}{\sin }^{2}\psi (z))}^{-1}dz}.$$

The integral requires a self-consistent solution for all the sublayers and makes calculations rather involved. We use a method of gradient descent to the free energy minimum. The approach is described in detail in ref.^8^.

In the first step we simulate the TN-FPC optical response taking the optical extinction and dispersion of materials into account. The case of normal light incidence is considered. The optical response is found using the Berreman method—the transfer-matrix method generalized for an anisotropic medium^33^. In the case of birefringent layered media, the electromagnetic radiation consists of four partial waves. Mode coupling takes place at the interface where an incident plane wave produces waves with different polarization states due to anisotropy of the layers. As a result, 4 × 4 matrices are required.
